# Questions to Measure Enjoyment of and Satisfaction With Physical Activity: Are They Appropriate for Use in an Older Population?

**DOI:** 10.1093/geroni/igab041

**Published:** 2021-10-04

**Authors:** Mary Katherine Huffman, Sharon L Christ, Kenneth F Ferraro, David B Klenosky, Kristine Marceau, Steve Amireault

**Affiliations:** 1 Department of Health and Kinesiology, Purdue University, West Lafayette, Indiana, USA; 2 Center on Aging and the Life Course, Purdue University, West Lafayette, Indiana, USA; 3 Department of Human Development and Family Studies, Purdue University, West Lafayette, Indiana, USA; 4 Department of Sociology, Purdue University, West Lafayette, Indiana, USA

**Keywords:** Exercise, Factor analysis, Measurement invariance, Surveys and questionnaires

## Abstract

**Background and Objectives:**

Enjoyment of and satisfaction with physical activity have been proposed as two actionable mechanisms to promote sustained engagement in physical activity. An accurate understanding of how, why, and for whom these two mechanisms work (or not) in response to a particular intervention strategy is contingent on having suitable measures for the population of interest. This study aims to determine whether the Physical Activity Enjoyment Scale-8 and a novel approach to the measurement of satisfaction with physical activity are suitable for use among older adults (*M*_age_ = 66.25 years; range = 55–91 years).

**Research Design and Methods:**

Participants answered an online questionnaire twice across 4 weeks. Measurement invariance was assessed within a structural equation modeling framework; convergent validity was assessed by correlating the latent variables enjoyment and satisfaction with each other and with physical activity behavior.

**Results:**

Both measures were invariant between gender and across time. Enjoyment and satisfaction were related to each other (*r* = 0.72) and to physical activity (*r* = 0.48 and 0.64, respectively).

**Discussion and Implications:**

Results support the suitability of these measures as tools to assess enjoyment of and satisfaction with physical activity among older adults.

**Translational Significance:** Enjoyment of and satisfaction with physical activity are two motives that may help older adults maintain their physical activity behaviors. We developed a brief, multi-item satisfaction measure and replicated previous findings supporting the validity of an enjoyment measure. These two measures can be used—either with latent variables or composite scale scores—to assess older adults’ motives for physical activity and to make direct comparisons across 4 weeks and between men and women.

Regular physical activity participation yields important health benefits in later life (Lachman et al., 2018). However, more than one quarter of adults 50 years or older report being physically inactive outside of work, and this prevalence increases with age (Watson et al., 2016). Although currently available physical activity programs can help older adults increase their physical activity, many struggle to continue engaging in this behavior beyond program completion (Sansano-Nadal et al., 2019). This is a significant public health challenge because the benefits of a single bout of physical activity are short-lived (e.g., a few minutes for improvements in cognition, a few hours for reductions in anxiety), and chronic adaptations to repeated exercise (e.g., improvement of cardiovascular and muscular functions) are lost within a few weeks of inactivity. According to behavioral maintenance theories (Kwasnicka et al., 2016; Nigg et al., 2008; Rothman, 2000), enjoyment of and satisfaction with physical activity are two key motivational levers for physical activity maintenance. Furthermore, there is emerging empirical evidence that these two motives underlie the maintenance of physical activity among older adults (Huffman et al., 2020; van Stralen et al., 2009). Therefore, enjoyment of and satisfaction with physical activity may represent key targets for interventions designed to promote program adherence and physical activity maintenance among older adults.

To determine whether enjoyment of and satisfaction with physical activity change over time in response to different intervention strategies, it is essential to have reliable measures that meaningfully capture these constructs within the population, within subgroups of the population of interest (e.g., men or women), and across time (Sheeran et al., 2017). However, the adequacies of score interpretations of common measures are often taken for granted without prior validation testing within the population of interest (Hagger & Chatsizarantis, 2009). Empirical evidence supporting the adequacies of score interpretations of enjoyment and satisfaction measures in the context of physical activity is limited (Chmielewski et al., 2016). The overall purpose of this study is to determine whether a measure of enjoyment of physical activity and a new measure of satisfaction with physical activity are suitable for use among an older population.

## Measurement Invariance

To enable the testing of relationships involving a given construct with other variables, it is essential to establish whether the chosen measure of the construct is invariant (Estabrook, 2012). Measurement invariance is comprised of configural invariance (i.e., equivalent forms of the model represented by the measure) and metric invariance (i.e., equivalent relationships between the scale items and the underlying construct; Schaie et al., 1998). When measurement invariance is established among subgroups of the population of interest, differences in relationships involving the construct can be attributed to subgroup differences. Similarly, a longitudinally invariant measure suggests that the meaning and interpretation of the underlying construct are the same when respondents are measured across different occasions. Without evidence for measurement invariance, observed differences between subgroups or changes in a construct over time may unknowingly be due to fluctuating interpretations of the scale items rather than true differences in the construct. If the interpretations of the scale items vary across subgroups of interest, but the scale is used as if it were invariant, estimated direct and indirect (i.e., mediation) effects may be inaccurate (Xu et al., 2020). This study investigates whether the interpretations of scale items for measuring enjoyment of and satisfaction with physical activity vary between men and women and across a 4-week timescale.

## Enjoyment of Physical Activity

Enjoyment of physical activity has been defined as a positive affective state brought about by engaging in the behavior itself (Wankel, 1993) or as an optimal psychological state that leads one to perform an activity primarily for its own sake (Kimiecik & Harris, 1996). People are more likely to choose to participate in physical activity during their discretionary time when it is perceived as being personally meaningful and immediately emotionally rewarding—that is, if it is perceived as enjoyable (Kwasnicka et al., 2016; Lachman et al., 2018; Wankel, 1993).

The 18-item Physical Activity Enjoyment Scale (PACES-18) was developed with samples of adults aged 18–65, and evidence of its validity was provided (Kendzierski & DeCarlo, 1991). Adapted versions of the PACES-18 have been assessed for invariance among children and adolescents (Dunton et al., 2009; Moore et al., 2009). However, the PACES-18 may not represent a well-fitting one-factor model for older adults (*M*_age_ = 66.43; Mullen et al., 2011). Thus, the PACES-8 was subsequently created (Mullen et al., 2011), and evidence of measurement invariance between two exercise groups (walking and flexing–toning–balance) and across a 6-month timescale was provided for this sample.

## Satisfaction With Physical Activity

Satisfaction with physical activity reflects a global assessment of the positive and negative experiences and outcomes derived from the behavior (Baldwin & Sala, 2018; Rothman, 2000). If more positive experiences (e.g., quality experiences with friends, feeling better during the activity) are perceived than negative ones (e.g., pain, fatigue), and if actual outcomes are similar to those initially expected and desired (e.g., improved functioning), motivation to continue physical activity is reinforced (Kwasnicka et al., 2016; Rothman, 2000).

Research on satisfaction with physical activity has often used a single-item measure (e.g., “In general, how satisfied are you with what you have experienced as a result of exercising?” on a rating scale that ranges from *extremely* or *very dissatisfied* to *extremely* or *very satisfied*; Baldwin et al., 2013; Chmielewski et al., 2016; Fleig et al., 2011). Although this single item closely reflects the underlying satisfaction construct, it is likely a suboptimal measurement approach for three main reasons. First, respondents tend to more frequently use the midpoint or positive (satisfied) end of the scale when compared with the negative (dissatisfied) end (Baldwin et al., 2013). It is unclear if this is because the wording of the question (i.e., “how *satisfied* are you …?”) leads respondents to focus only on satisfaction, or because of people’s tendency to provide positive ratings when answering questions on satisfaction (Choi & Pak, 2005), or both. Second, this single item has been reported as having suboptimal psychometric properties, including evidence of weak test–retest reliability and validity (Chmielewski et al., 2016). Third, perceived satisfaction is theorized as a multifaceted construct (Baldwin & Sala, 2018), which may not be adequately appraised using one question. To improve item content relevance and representation of the satisfaction construct and to safeguard against potential reporting issues, we developed a new multi-item measure of satisfaction with physical activity.

## Objectives

The purpose of this study was to determine whether the PACES-8 and a novel approach for measuring satisfaction with physical activity are suitable for use among older adults (aged ≥55 years). Specifically, we examined measurement invariance between men and women and across two measurement occasions 4 weeks apart. We also quantified the associations between the enjoyment and satisfaction measures and between these two maintenance motives and physical activity behavior.

This study contributes to the physical activity and gerontology literature in three important ways. First, this study reports the development, validity, and reliability of a new multi-item measure of satisfaction with physical activity. This new measure was developed with members of the older population to create a relatively brief scale that better represents the multifaceted nature of satisfaction with physical activity. Second, this study constitutes an attempt to replicate Mullen et al.’s (2011) findings regarding the structural validity (evidence for a single-factor model) of the PACES-8 for use with older adults. This is relevant because the rejection of the PACES-18 and the creation of the PACES-8 were originally tested within the same sample (Mullen et al., 2011). Third, this study establishes the suitability of these two measures by testing invariance between men and women and across a 4-week timescale. Gender invariance was examined, as sex stereotypes and gender roles may affect how people perceive their motivations for and value ascribed to their physical activity (Chalabaev et al., 2013; Semerjian, 2018). Additionally, Mullen et al. (2011) determined the PACES-8 was invariant across 6 months. Because experiences and outcomes that occur within the first few weeks after one’s change in his or her behavior are linked to satisfaction with physical activity (Baldwin et al., 2013; Chmielewski et al., 2016), a 4-week (i.e., about 1 month) timescale was chosen to assess longitudinal invariance.

## Research Design and Methods

### Participants and Procedures

**Figure 1.**
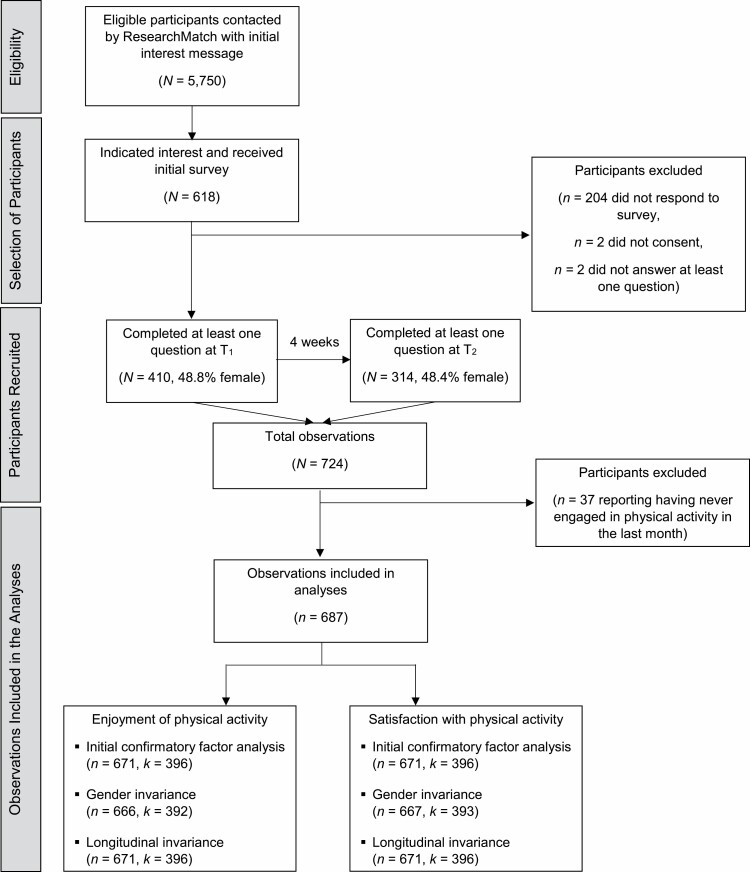
Participant flowchart. *Notes: N* = number of respondents; *n* = number of observations; *k* = number of clusters. Percentage of female responses for T_1_ and T_2_ was calculated after missing gender information was imputed based on available responses from the prior or subsequent time point.

Older adults living in the United States were recruited through ResearchMatch (www.researchmatch.org), a national online health research volunteer registry that was created by several academic institutions and supported by the U.S. National Institutes of Health as part of the Clinical Translational Science Award program. ResearchMatch has a large population of volunteers who have consented to be contacted by researchers about health studies for which they may be eligible. To be eligible for the current study, participants were required to be at least 55 years of age, be able to read and understand English, and to have no indication of cognitive impairment. Previous research has suggested the use of the age group 55–64 as a benchmark denoting age-related declines in health (Schoenborn & Heyman, 2009). We nonetheless acknowledge that defining the older adult population as 55 years of age or older is somewhat arbitrary. Potential participants were identified by filtering on these eligibility criteria in ResearchMatch’s participant selection system, and an initial message detailing the study was sent in batches to randomly selected individuals meeting the criteria. As one purpose of this study is to test the measurement invariance between genders, the initial interest message was purposefully sent in gender batches to attempt to recruit equal numbers of men and women. In total, 5,750 older adults were randomly selected by ResearchMatch’s participant selection system and were sent the interest message. Those indicating interest were then emailed a unique link to an online Qualtrics survey. After clicking “I agree” to an online consent form, they completed the first survey (T_1_). Four weeks later (T_2_), the participants received an email inviting them to take a second identical survey. Data collection occurred from August to September 2019. A participant flowchart is presented in Figure 1. Participants were compensated with a $10 Amazon gift card. This study was approved by the Purdue University Institutional Review Board (IRB Protocol #: 1906022325). See Supplementary Section A for further information regarding recruitment and sample size.

### Measures

#### Enjoyment of physical activity

The PACES-8 (Mullen et al., 2011) consists of eight items and asks participants to rate how they feel at the moment about the physical activity they have been doing. Responses were indicated on a 7-point scale and included choices such as “I find it pleasurable/I find it unpleasurable.” Six items were reverse-coded such that higher scores on the PACES-8 indicated more enjoyment. The full scale is presented in Supplementary Section B.

#### Satisfaction with physical activity

The satisfaction with physical activity measurement approach was developed following a three-step process with a separate sample of 10 older adults (six males, 77–85 years; IRB Protocol #: 1902021741). Further details of the three-step development process are presented in Supplementary Section C. Briefly, the single item that has commonly been used in past research was retained, as it closely reflects the theoretical definition of the satisfaction construct (Baldwin & Sala, 2018; Rothman, 2000). We slightly modified this item by adding the word “dissatisfied” to the question (i.e., “As of today, how dissatisfied or satisfied are you with what you have experienced as a result of regularly engaging in physical activity?”). The developed set of scale items includes three additional items that tap onto different facets of satisfaction, namely *expectancy violation*, *realizations given the expended effort*, and *emotional responses*. Responses are indicated on a 7-point scale (e.g., *very dissatisfied* to *very satisfied*). The four-item measure is fully presented in Supplementary Section C.

#### Leisure-time physical activity

A one-item measure of physical activity behavior was used to assess how often in the past month the participants had been physically active for at least 30 min on the same day (Godin et al., 1986). Responses ranged from 1 (*never*) to 7 (*4 days or more per week*). Prior to answering the question, the following definition of physical activity was provided to respondents: “Physical activity refers to activities that get your body moving. Doing such activities would result in noticeable increases in breathing, heart rate, or sweating.” Consistent with recommendations to measure physical activity in older adults (Rikli, 2000; Sattler et al., 2020), examples of activities that older adults typically engage in (e.g., gardening, walking; Amireault et al., 2019; DiPietro, 2001) were provided. Participants were explicitly instructed not to include activities that they engaged in as part of their job, volunteering, or caretaker duties. This measure was selected because it does not ask directly about physical activity intensity or duration, which may contribute to mitigate measurement error associated with the reporting of physical activity (Rikli, 2000). Correlations between the number of days of being physically active for ≥30 min and accelerometry measures (Milton et al., 2013; Wanner et al., 2014) and fitness center visit frequency (weekly number of mandatory check-ins or card swipes; Amireault & Godin, 2014) ranged from 0.44 to 0.57 for the adult population aged 18 and older.

#### Sample demographic and health characteristics

Participants self-reported their age, gender, race/ethnicity, education, employment, relationship status, self-rated health, and any chronic conditions (e.g., arthritis) experienced. Weight and height were self-reported to calculate body mass index (BMI). BMI was calculated by dividing weight (kilograms) by height squared (m^2^).

#### Survey administration

Eligible participants who were sent the initial interest message via ResearchMatch were given the option to decline participation in the study or release email address contact information to the researchers. Those who released this information were emailed a unique link to an online Qualtrics survey that could only be used once. After clicking “I agree” to an online consent form, participants completed the first survey (T_1_). Responses were automatically saved while the participants were taking the survey, and they could go back to change answers or finish at a separate time. The physical activity question was consistently asked first, and the demographics and health characteristics questions were consistently asked last. The order of the PACES-8 and the satisfaction measure was randomized. Moreover, the items within each measure were also randomized such that they did not appear in a consistent order across participants. Randomization was implemented using Qualtrics’ randomization features. Four weeks later (T_2_), the participants received an email inviting them to take a second identical survey. Out of 618 older adults who released their contact information and received a T_1_ survey link, 410 consented and answered at least one question; at T_2_, 314 of the 410 participants answered at least one question (Figure 1; see also Supplementary Section A, Figure A.1 for a study timeline).

### Data Analysis

#### Data screening and preparation

First, data were screened for out-of-range values, missing data, and distributional anomalies. As both the enjoyment and satisfaction measures utilize Likert-type responses, outliers were not considered an issue. A comparison was made between those who responded to the survey at both times and those who dropped out after completing the survey at T_1_. There was no evidence of demographic differences, and the two groups were largely similar. Supplementary Section D reports more details regarding data preparation and the comparison between dropouts and completers. Additionally, because the PACES-8 asks about “the physical activity you have been doing,” and the satisfaction with physical activity measure asks individuals to assess their satisfaction with experiences and outcomes derived from past physical activity, those indicating that they have not participated in physical activity in the last month were excluded (*n* = 37). Data screening was conducted using SAS version 9.4 (SAS Institute Inc., Cary, NC) and Stata version 16 (StataCorp LLC, College Station, TX).

#### Gender and longitudinal invariance analyses

The invariance analyses were conducted using Stata version 16 (StataCorp LLC). Measurement invariance was assessed within a structural equation modeling framework (Bollen, 1989). First, a one-factor model was specified for both enjoyment and satisfaction, and model fit was assessed using confirmatory factor analysis for the entire sample. Second, configural invariance was tested to determine whether the form (e.g., number of underlying latent variables) was the same between men and women and also across T_1_ and T_2_ when allowing parameter estimates to be freely estimated (i.e., different between gender and time groups). Finally, to test metric invariance, factor loadings were constrained to be equal between men and women and across T_1_ and T_2_ to determine whether the relationships between the latent variables and the indicators were the same. Lagrange multiplier tests were performed to determine whether the factor loadings were the same for both men and women and at both measurement occasions. Given the multiple tests run, an alpha value of 0.01 was used as the significance level for the Lagrange multiplier tests.

 Observations were pooled from both T_1_ and T_2_ for the analyses. To account for this clustering of repeated measures within respondents, cluster variance estimation was used. Moreover, because the distributions of the enjoyment and satisfaction item scores were negatively skewed (Supplementary Section D), clustered bootstrapping with 500 replications was used to obtain standard errors to correct for nonnormal outcomes for the initial confirmatory factor analyses and to assess invariance of form. Observations were listwise deleted automatically when the bootstrap procedure was used. The robust cluster estimator was used to assess invariance of factor loadings due to the incompatibility of Stata invariance commands with bootstrap results. Figure 1 provides the number of observations (*n*) and clusters (*k*) for all analyses. The invariance analyses were run with a data set that included those who responded “never” to the physical activity item. Overall results did not change.

Multiple model fit criteria were examined when considering how well the models fit the data. Global fit measures include the chi-square test (χ ^2^; *p* > .05 for acceptable fit), the Tucker–Lewis Index (TLI; ≥.90 for acceptable fit), the comparative fit index (CFI; ≥.90 for acceptable fit), the root mean square error of approximation (RMSEA; <.10 for acceptable fit), the coefficient of determination (CD; ≥.90 for acceptable fit), and the standardized root mean square residual (SRMR; <.08 for acceptable fit). Component fit measures (i.e., factor loadings, reliability values [*R*^2^]) were also considered. The *R*^2^ value represents a structural equation approach to item reliability that can be interpreted as the proportion of variance in a scale item that is explained by the underlying construct (Bollen, 1989). Although ranges and thresholds for satisfactory reliability scores are somewhat arbitrary, *R*^2^ values ≥0.70 were considered strong, values >0.40 and <0.70 were considered moderate, and values ≤0.40 were considered weak (Bollen, 1989). Internal consistency reliability (i.e., the extent to which multiple items measure the same underlying construct) was evaluated using Omega coefficients (McDonald, 1999).

#### Validity evidence based on relations to other variables

The models were used to assess the correlation between the latent variables of enjoyment and satisfaction. The correlations between the latent variables and the physical activity measure were also estimated and reported. Because enjoyment of and satisfaction with physical activity are overlapping yet conceptually distinct constructs (Baldwin & Sala, 2018; Chmielewski et al., 2016), they should be strongly positively correlated. Past studies have reported correlations of 0.38 and 0.57 between enjoyment of physical activity based on the PACES-18 and satisfaction with physical activity based on both a one-item measure of overall satisfaction (Chmielewski et al., 2016) and an expectancy violation measure (Williams et al., 2008). Given the satisfaction measurement approach developed in this study, the correlation reported here was expected to be stronger. It also was hypothesized that enjoyment of and satisfaction with physical activity would be strongly and positively correlated with physical activity behavior. Past research has reported correlations ranging from 0.15 to 0.27 between enjoyment and physical activity (Chmielewski et al., 2016; Mullen et al., 2011; Williams et al., 2008) and from 0.17 to 0.33 between satisfaction and physical activity (Chmielewski et al., 2016; Fleig et al., 2011; Williams et al., 2016). The correlations reported in this study were expected to be stronger than what has been found in past research due to the removal of random measurement error using structural equation modeling.

## Results

The participants reporting being physically active at least once in the last month were on average 66.25 years of age (*SE* = 0.37; range = 55–91 years), with an average BMI of 27.82 (*SE* = 0.29) at T_1_. Additional participant demographics are presented in Supplementary Section E, Table E.1. Descriptive statistics for the PACES-8 and satisfaction items at both T_1_ and T_2_ are presented in Supplementary Section E, Table E.2. Respondents participated in physical activity between 2 and 3 days per week at both measurement occasions (T_1_: *M* = 5.81, *SE* = 0.07; T_2_: *M* = 5.69, *SE* = 0.09; scale items ranging from 2 [*about once in the last month*] to 7 [*4 days or more per week*]).

### Enjoyment of Physical Activity

The enjoyment model was specified such that all eight items were indicators of enjoyment, error covariances were set to be 0, and enjoyment was scaled to the first item (i.e., “Pleasurable”). Model fit was then assessed for the entire data set. The model demonstrated an adequate fit, as all global model fit statistics were acceptable (TLI = 0.944, CFI = 0.960, CD = 0.941, SRMR = 0.032) except the chi-squared statistic (183.948, *df* = 20, *p* < .05) and the RMSEA value (0.111, 90% CI: [0.096, 0.126]). All factor loadings were significant, and item reliability values were moderate to large, ranging from 0.54 to 0.73. The model was respecified to improve fit before invariance testing. The first three items of the PACES-8 are phrased such that they elicit opinions of the physical activity experience itself (e.g., finding physical activity fun or pleasurable), whereas the remainder of the items are phrased such that respondents may reflect upon their positive affect after participation (e.g., feeling invigorated or stimulated). Thus, the model was respecified such that the errors of the first three items were allowed to correlate. This model demonstrated a better global fit to the data (χ ^2^ = 48.768, *df* = 17, *p* < .05; TLI = 0.987; CFI = 0.992; RMSEA = 0.053, 90% CI: [0.036, 0.070]; CD = 0.929; SRMR = 0.017) and a similar component fit to the data; therefore, this model was retained for the invariance analyses. Omega coefficients ranged between 0.93 and 0.95 (Supplementary Section E, Table E.3.).

#### Gender invariance

The form of the respecified model (i.e., the model with correlated error terms) was tested for men and women to determine whether the form was the same for both genders. The model fit well for both groups. Regarding component fit, all factor loadings were significant and in the same direction, and item reliability values were moderate to large (Table 1). Global fit measures also indicated a good fit (χ ^2^ = 66.717, *df* = 34, *p* < .05; TLI = 0.987; CFI = 0.992; RMSEA = 0.054, 90% CI: [0.034, 0.073]; CD = 0.930; SRMR = 0.021). Factor loadings were then constrained to be equal across genders. Results from the Lagrange multiplier tests were all nonsignificant (Table 2), indicating that all factor loadings were the same for men and women. Additionally, results from the Wald tests of equal covariances across gender were nonsignificant, indicating that the covariances among the three error terms were the same for men and women.

**Table 1.** Component Fit Indices for the Enjoyment and Satisfaction Models With Different Estimates Across Groups—Gender Analyses

Item No.	Parameter	Coefficient		Bootstrap 95% confidence interval		*R* ^2^ values	
		Males	Females	Males	Females	Males	Females
	*Enjoyment*						
1	Pleasurable	1 (constrained)		—	—	0.62	0.68
2	Fun	1.05	0.98	0.94, 1.16	0.84, 1.12	0.60	0.60
3	Pleasant	1.00	0.96	0.90, 1.09	0.84, 1.07	0.55	0.62
4	Invigorating	1.15	0.91	1.01, 1.28	0.73, 1.09	0.76	0.63
5	Gratifying	1.02	0.88	0.86, 1.19	0.76, 1.01	0.64	0.58
6	Exhilarating	1.13	1.06	0.98, 1.27	0.92, 1.20	0.70	0.68
7	Stimulating	1.04	0.89	0.86, 1.22	0.76, 1.01	0.57	0.54
8	Refreshing	1.18	1.08	1.06, 1.31	0.96, 1.20	0.76	0.73
	cov(1, 2)	0.21	0.17	0.10, 0.32	0.05, 0.29		
	cov(1, 3)	0.36	0.21	0.22, 0.49	0.10, 0.32		
	cov(2, 3)	0.30	0.23	0.16, 0.45	0.11, 0.36		
	*Satisfaction*						
1	**Evaluation**	1 (constrained)		—	—	0.74	0.74
2	Expectations	0.72	0.72	0.60, 0.84	0.59, 0.86	0.50	0.46
3	Realizations	1.05	1.09	0.91, 1.18	0.95, 1.23	0.81	0.81
4	Emotion	0.87	0.87	0.77, 0.98	0.75, 0.99	0.74	0.77

*Notes:* All coefficients are significant (*p* < .05). “cov(*x*, *y*)” indicates the covariance between item *x* and item *y*. *R*^2^: reliability values. The bolded satisfaction item represents the commonly used single-item measure of satisfaction.

**Table 2.** Invariance Results for the Enjoyment and Satisfaction Models With Equal Factor Loadings Across Groups

Item No.	Parameter	Gender invariance		Longitudinal invariance	
		χ ^2^	*p*	χ ^2^	p
	*Enjoyment*				
1	Pleasurable	2.05	.15	2.52	.13
2	Fun	0.01	.91	0.01	.91
3	Pleasant	0.20	.65	1.05	.31
4	Invigorating	4.20	.04	0.07	.79
5	Gratifying	0.16	.69	2.02	.16
6	Exhilarating	0.59	.44	1.99	.16
7	Stimulating	0.27	.60	0.45	.50
8	Refreshing	0.10	.75	0.63	.43
	cov(1, 2)	0.06	.81	0.10	.75
	cov(1, 3)	1.85	.17	0.07	.80
	cov(2, 3)	0.28	.60	0.46	.50
	*Satisfaction*				
1	**Evaluation**	0.06	.81	0.05	.83
2	Expectations	0.01	.91	0.22	.64
3	Realizations	0.30	.59	0.11	.74
4	Emotion	0.07	.79	0.02	.89

*Notes:* The null hypothesis of the Lagrange multiplier test is that the constraint (i.e., constraining the factor loading to be equal across groups) is valid. Lagrange multiplier test results are reported for parameters that were constrained (i.e., the factor loadings). The null hypothesis of the Wald test is that a constraint would have been valid. Wald test results are reported for parameters that were not constrained (i.e., the error covariances). “cov(*x*, *y*)” indicates the covariance between item *x* and item *y*. The bolded satisfaction item represents the commonly used single-item measure of satisfaction.

#### Longitudinal invariance

The form of the respecified model was tested across T_1_ and T_2_. The model fit well at both measurement occasions (χ ^2^ = 61.715, *df* = 34, *p* < .05; TLI = 0.989; CFI = 0.993; RMSEA = 0.049, 90% CI: [0.029, 0.069]; CD = 0.930; SRMR = 0.018). Factor loadings were all significant and in the same direction, and item reliability values were moderate to large (Table 3). Factor loadings were constrained to be equal at both time points, and results from the Lagrange multiplier tests were nonsignificant (Table 2), indicating that factor loadings were the same at both times. Results from the Wald tests of equal covariances across time were also nonsignificant, indicating stable covariances across time.

**Table 3.** Component Fit Indices for the Enjoyment and Satisfaction Models With Different Estimates Across Groups—Longitudinal Analyses

Item No.	Parameter	Coefficient		Bootstrap 95% confidence interval		*R* ^2^ values	
		Time 1	Time 2	Time 1	Time 2	Time 1	Time 2
	*Enjoyment*						
1	Pleasurable	1 (constrained)		—	—	0.66	0.62
2	Fun	0.98	1.05	0.88, 1.09	0.92, 1.19	0.59	0.61
3	Pleasant	0.94	1.04	0.84, 1.04	0.94, 1.15	0.56	0.62
4	Invigorating	1.00	1.09	0.88, 1.12	0.93, 1.25	0.71	0.69
5	Gratifying	0.97	0.93	0.85, 1.09	0.78, 1.09	0.64	0.57
6	Exhilarating	1.04	1.19	0.91, 1.16	1.03, 1.35	0.67	0.73
7	Stimulating	0.97	0.99	0.83, 1.11	0.85, 1.13	0.58	0.54
8	Refreshing	1.08	1.20	0.99, 1.17	1.07, 1.33	0.72	0.78
	cov(1, 2)	0.19	0.22	0.07, 0.30	0.11, 0.33		
	cov(1, 3)	0.29	0.31	0.16, 0.41	0.20, 0.43		
	cov(2, 3)	0.30	0.24	0.15, 0.44	0.13, 0.35		
	*Satisfaction*						
1	**Evaluation**	1 (constrained)		—	—	0.74	0.74
2	Expectations	0.71	0.73	0.60, 0.83	0.61, 0.85	0.47	0.50
3	Realizations	1.08	1.05	0.94, 1.22	0.93, 1.17	0.79	0.83
4	Emotion	0.88	0.87	0.77, 0.99	0.76, 0.97	0.73	0.78

*Notes:* All coefficients are significant (*p* < .05). “cov(*x*, *y*)” indicates the covariance between item *x* and item *y*. *R*^2^: reliability values. The bolded satisfaction item represents the commonly used single-item measure of satisfaction.

### Satisfaction With Physical Activity

The satisfaction model was specified such that all four items were indicators of satisfaction, error covariances were set to be 0, and satisfaction was scaled to the first item (i.e., “Evaluation”). The fit of the model for the entire data set was assessed. The model demonstrated an excellent overall fit. All global model fit statistics were acceptable (χ ^2^ = 4.214, *df* = 2, *p* > .05; TLI = 0.996; CFI = 0.999; RMSEA = 0.041, 90% CI: [0.000, 0.096]; CD = 0.917; SRMR = 0.008), factor loadings were all significant, and item reliability values were moderate to large, ranging from 0.48 to 0.81. Omega coefficients ranged between 0.89 and 0.91 (Supplementary Section E, Table E.3.).

#### Gender invariance

The form of the model was then tested for men and women. The global model fit was excellent for both groups (χ ^2^ = 5.562, *df* = 4, *p* > .05; TLI = 0.997; CFI = 0.999; RMSEA = 0.034, 90% CI: [0.000, 0.095]; CD = 0.918; SRMR = 0.010). Additionally, all factor loadings were significant and in the same direction, and item reliability values were moderate to large (Table 1). Factor loadings were constrained to be equal for men and women, and the results of the Lagrange multiplier tests were all nonsignificant (Table 2); thus, factor loadings were the same between genders.

#### Longitudinal invariance

The form of the model was tested across time, and the model fit demonstrated an excellent fit at both times (χ ^2^ = 4.607, *df* = 4, *p* > .05; TLI = 0.999; CFI = 1.000; RMSEA = 0.021, 90% CI: [0.000, 0.088]; CD = 0.916; SRMR = 0.008). Factor loadings were all significant and in the same direction, and item reliability values were moderate to large (Table 3). Finally, factor loadings were constrained to be equal at T_1_ and T_2_. The Lagrange multiplier tests were all nonsignificant (Table 2).

### Validity Evidence Based on Relations to Other Variables

**Figure 2.**
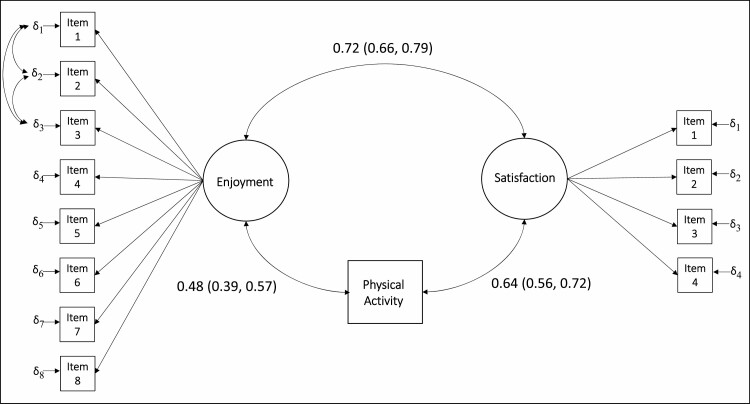
Correlations between latent variables and physical activity behavior. *Notes: n* = 664, *k* = 395. δ denotes the item errors. All *p* values ≤.001. Analysis was conducted using clustered bootstrapping with 500 replications. *n* = number of observations; *k* = number of clusters.

Figure 2 indicates that the latent variables of the invariant models were positively correlated with the physical activity measure and with each other. All correlations were positive and stronger in magnitude compared to those reported by prior research.

## Discussion and Implications

The purpose of this study was to determine whether the PACES-8 and a novel approach to the measurement of satisfaction with physical activity were suitable for use among older adults (aged ≥55 years). Past research has explicitly highlighted the need for the development of a new approach to the measurement of satisfaction within the physical activity literature (Chmielewski et al., 2016). The measurement instrument used in this study was purposefully developed with a sample of older adults to address this need. The measurement approach evaluated in this study includes multiple items to assess the multifaceted construct of satisfaction and phrases questions in a way that is less prone to lead respondents to focus only on the positive end of the scale. Moreover, this novel, four-item measure was found to represent a well-fitting, one-factor model and to be invariant between men and women and across two measurement occasions 4 weeks apart. Additionally, the latent variable satisfaction with physical activity was positively correlated with physical activity behavior (*r* = 0.64) and with the latent variable enjoyment (*r* = 0.72), and the measure had high reliability (omega coefficients) at both measurement occasions for both men and women. Thus, this study provides evidence for a psychometrically sound, brief multi-item satisfaction measure for use with older adults in a physical activity context.

Previously, Mullen et al. (2011) found that the PACES-8 represented a well-fitting, one-factor model of enjoyment and was invariant between two exercise groups and across two measurement occasions, 6 months apart, for a sample of older adults (*M*_age_ = 66.43). Consistent with these results, this study provides additional support for a one-factor model among an older adult sample. The current study also demonstrated that the PACES-8 is invariant between gender (men and women) and across a shorter timeframe (4 weeks), further establishing its robustness within the older population. Moreover, compared to the correlations reported in Mullen et al.’s (2011) study (*r* = 0.16 and 0.17), a stronger positive correlation between the latent variable enjoyment and physical activity behavior was calculated in the current study (*r* = 0.48). It should be noted that the smaller correlations between enjoyment and physical activity found by Mullen et al. (2011) may be due to the fact that the Physical Activity Scale for the Elderly (Washburn et al., 1993) used in the prior study assesses domains in which the physical activity performed may not be perceived as enjoyable (e.g., occupational activity). These domains were specifically excluded from the physical activity measure used in the current study. Additionally, Mullen et al. (2011) calculated their correlation with the total scale score rather than the latent construct enjoyment.

Often in the physical activity and health psychology literature, items from Likert-type scales are summed or averaged to create a composite score to use as a predictor or outcome variable. Importantly, however, if the relations between the indicators and construct of interest differ between groups (or across time), summing or averaging the scores of scale items in the same way for different groups (or at several time points) would provide estimates of the construct that are not directly comparable. This study revealed that factor loadings could be considered equal in all invariance analyses, suggesting that the relationships between the PACES-8 and satisfaction items and the latent variables enjoyment of and satisfaction with physical activity, respectively, are interpreted similarly between men and women and across a 4-week timescale. Thus, creating a composite score in this way for these groups would be acceptable. This study also found that the better-fitting model for enjoyment included correlated errors between the first three items of the PACES-8. A strength of structural equation modeling is that it can account for this measurement error; other methods (e.g., multiple linear regression) assume that variables are measured error-free. While these correlated errors do not affect how the scale associates with other variables, researchers may wish to utilize a structural equation modeling framework when measuring enjoyment of physical activity with the PACES-8 and account for these correlated errors in order to use a better fitting model.

### Limitations

The generalizability of the study findings is limited by the underrepresentation of certain subgroups of the older adult population. The sample consisted predominately of educated, White individuals who were relatively younger, considering the broad age range of the older population. Additionally, the participants included in the invariance and validity analyses were preregistered members of an existing national online health research volunteer registry, and therefore, results may not generalize to older adults who are less interested in health research. Moreover, respondents were limited to older adults living in the United States. It is thus likely that most participants of the study could be considered as having a Western cultural background. Enjoyment and satisfaction may have different meanings for individuals of other cultures. Lastly, the self-reported data may be subject to social desirability bias and shared method variance. This may have inflated the correlations between enjoyment, satisfaction, and physical activity.

## Conclusions

Enjoyment of and satisfaction with physical activity represent two theoretical constructs that may facilitate older adults’ sustained engagement in physical activity. These constructs were assessed using the PACES-8 and a new approach to the measurement of satisfaction with physical activity. Notably, this new satisfaction measurement approach consists of a relatively brief, four-item measure that better represents the multifaceted nature of the satisfaction construct. We conclude that these measures are suitable for use among adults aged 55 and older and should therefore be used in future research investigating these constructs among this population.

## Supplementary Material

igab041_suppl_Supplementary_Materials

## Author Note

The study was carried out at Purdue University. M. K. Huffman is now at the Department of Pharmaceutical Health Services Research, University of Maryland, Baltimore, USA.
