# Supplementary material for: Questions to Measure Enjoyment of and Satisfaction With Physical Activity: Are They Appropriate for Use in an Older Population?
Source: Innov Aging. 2021 Oct 4;5(4):igab041. doi: 10.1093/geroni/igab041 (PMC8713737; doi:10.1093/geroni/igab041)
Supplement: igab041_suppl_Supplementary_Materials [file igab041_suppl_supplementary_materials.docx]

ONLINE SUPPLEMENTARY MATERIAL

Section A.

Recruitment, Sample Size, and Study Timeline

Prior to conducting this study, we did not know how many potential participants to expect to respond to the initial ResearchMatch message with interest. Moreover, as we sought to recruit equal numbers of both genders, we were cautious not to unintentionally recruit a disproportional number of men to women. Therefore, we proceeded to send the initial message in gender batches of 200 - 400 and noted in real time the respective numbers of men and women who responded with interest. We subsequently sent more batch messages when more participants were required for a gender. Within a structural equation modeling framework, it is recommended to have at least “several cases” per free parameter estimated (Bollen, 1989). Our enjoyment model (the largest of the two models) required the estimation of 16 and 19 parameters for the initial and re-specified model, respectively, for the first confirmatory factor analysis. We therefore attempted to recruit and retain 200 men and 200 women for a total of 400 participants at each measurement occasion. A total number of 800 observations thus would have allowed for 50 and 42 cases per parameter for the initial and re-specified enjoyment model, respectively. Even with a conservative number of 600 observations, the confirmatory factor analyses would still have had “several” cases per free parameter.

**Reference**

Bollen, K. A. (1989). *Structural equations with latent variables*. John Wiley & Sons. https://doi.org/10.2307/2072165


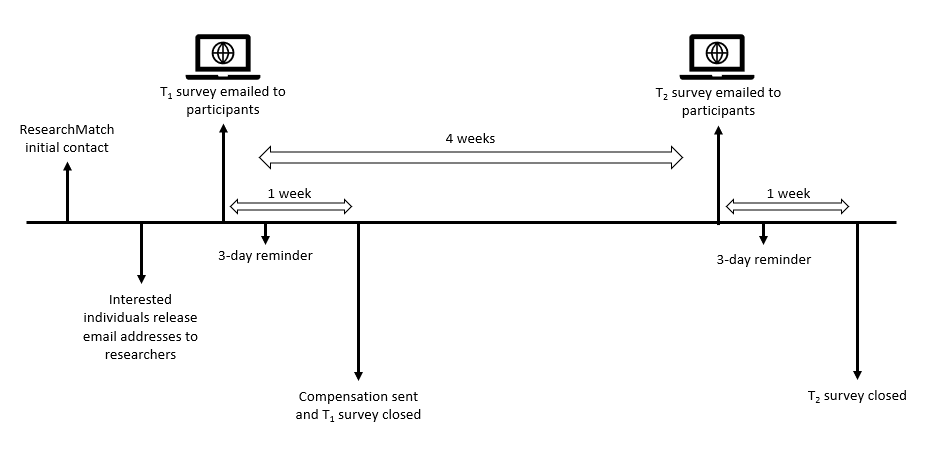


Supplementary Figure A.1. Study timeline from August 2019 to September 2019.

Section B.

Physical Activity Enjoyment Scale-8 (items 1 to 4)

Please rate how you feel at the moment about the physical activity you have been doing.

| 1 | - I find it pleasurable | - I find it mostly pleasurable | - I find it slightly pleasurable | Neutral | - I find it slightly unpleasurable | - I find it mostly unpleasurable | - I find it unpleasurable |
| --- | --- | --- | --- | --- | --- | --- | --- |
| 2 | - It's no fun at all | - It's mostly no fun | - It's slightly no fun | Neutral | - It's slightly fun | - It's mostly fun | - It's a lot of fun |
| 3 | - It's very pleasant | - It's mostly pleasant | - It's slightly pleasant | Neutral | - It's slightly unpleasant | - It's mostly unpleasant | - It's very unpleasant |
| 4 | - It's very invigorating | - It's mostly invigorating | - It's slightly invigorating | Neutral | - It's slightly not invigorating | - It's mostly not invigorating | - It's not at all invigorating |

Physical Activity Enjoyment Scale-8 (items 5 to 8)

Please rate how you feel at the moment about the physical activity you have been doing.

| 5 | - It's very gratifying | - It's mostly gratifying | - It's slightly gratifying | Neutral | - It's slightly not gratifying | - It's mostly not gratifying | - It's not at all gratifying |
| --- | --- | --- | --- | --- | --- | --- | --- |
| 6 | - It's very exhilarating | - It's mostly exhilarating | - It's slightly exhilarating | Neutral | - It's slightly not exhilarating | - It's mostly not exhilarating | - It's not at all exhilarating |
| 7 | - It's not at all stimulating | - It's mostly not stimulating | - It's slightly not stimulating | Neutral | - It's slightly stimulating | - It's mostly stimulating | - It's very stimulating |
| 8 | - It's very refreshing | - It's mostly refreshing | - It's slightly refreshing | Neutral | - It's slightly not refreshing | - It's mostly not refreshing | - It's not at all refreshing |

Section C.

Satisfaction Measure

When you think about engaging in physical activity, you probably have some expectations about the physical activity experience itself (i.e., feelings while doing it) and its consequences (i.e., benefits from it). The following questions will ask you to describe your feeling of satisfaction with your physical activity.

1. As of today, how dissatisfied or satisfied are you with what you have experienced as a result of regularly engaging in physical activity?

- Very dissatisfied
- Dissatisfied
- Slightly dissatisfied
- Neutral
- Slightly satisfied
- Satisfied
- Very satisfied

1. To what extent do you think your expectations for regularly engaging in physical activity in the last month have been realized?

- Very much worse than expected
- Worse than expected
- Slightly worse than expected
- Matched expectations
- Slightly better than expected
- Better than expected
- Very much better than expected

1. Given the effort you put into trying to meet your physical activity goals in the last month, how would you rate the outcomes (e.g., benefits and feelings) of physical activity you have experienced?

- Very poor
- Poor
- Slightly poor
- Fair
- Slightly good
- Good
- Very good

1. How would you describe your feeling of satisfaction with what you have experienced as a result of regularly engaging in physical activity in the last month?

- Terrible
- Unhappy
- Slightly unhappy
- Neutral
- Slightly pleased
- Pleased
- Delighted

Three-Step Development Process for a New Measure of Satisfaction

**Step 1: Review of the literature**

Expectancy violation, realizations given the expended effort, and emotional responses were three facets of the satisfaction construct determined through a review of the health psychology, physical activity psychology, and consumer sciences literature.

*Expectancy violation*. Satisfaction includes an assessment of expectancy violation (e.g., Sears & Stanton, 2001; Williams et al., 2008) – that is, whether initial expectations were not achieved. Consistent with theory and this facet of satisfaction, older adults who initially expected to improve their physical fitness, weight, and body appearance – but perceived no or lower than expected improvements in these outcomes – demonstrated lower physical activity participation compared to those whose perceived outcomes better matched their expectations (Jones et al., 2005; Neff & King, 1995; Wilcox et al., 2006).

*Realizations given the expended effort*. A relative assessment of the costs and benefits from engaging in a behavior is theorized to inform people’s overall satisfaction assessment (Rothman, 2000). This facet can be reflected in one’s rating of the outcomes afforded by the exercise or physical activity experience, given the effort one put into trying to reach or meet relevant personal goals (e.g., Baldwin et al., 2009; Williams et al., 2016).

*Emotional responses*. Building upon consumer sciences and marketing literature, intense negative or positive emotional responses to an experience may consist of high levels of dissatisfaction or satisfaction stemming from elements of surprise. For instance, people might be disgusted (extreme dissatisfaction) or delighted (extreme satisfaction) when they perceive their performance to surprisingly fall well below or far exceed one’s initial expectations, respectively (Barnes & Krallman, 2019; Ma et al., 2017). Consistent with this view, older women who did not initially expect to improve their physical fitness and reduce their stress but perceived improvements six months later (“surprised pessimists”) reported higher physical activity levels compared to those whose initial expectations for change in fitness and stress were high but perceived no improvements (“disappointed optimists”) (Neff & King, 1995; Wilcox et al., 2006).

**Step 2: Draft of the satisfaction measure**

The new measure of satisfaction was drafted. The single item that has commonly been used in past research was retained, as it closely reflects the theoretical definition of perceived satisfaction (Baldwin & Sala, 2018; Rothman, 2000). However, the wording of the question was modified so that it was less likely to lead the respondents to focus only on the positive end (i.e., “As of today, how dissatisfied or satisfied are you…?” instead of “As of today, how satisfied are you…?”). One question for each of the other three relevant facets of perceived satisfaction identified in the review of the literature was created on a 5-point bipolar scale.

**Step 3: Cognitive interviews**

The purpose for conducting the cognitive interviews was to identify potential problems related to communicating the intent of the meaning of the questions to respondents, verify whether respondents were likely to not know or have trouble remembering information, and assess the adequacy of the range of responses to be recorded. A total of 10 face-to-face interviews were conducted with six older males and four older females (77 to 85 years of age) from the surrounding community area after providing their consent (IRB Protocol #: 1902021741). The interviewees were well educated (all completed a university degree) and primarily white/Caucasian. A verbal, retrospective probing approach was used (Willis, 2004). Accordingly, after each participant completed all satisfaction items, a trained interviewer asked questions to probe for information related to the clarity of the questions and adequacy of the response categories. All participants shared a similar understanding of “satisfaction” and “expectations” that was consistent with theory and the intent of the measure. Finally, interview data suggested that the participants could appropriately discriminate response categories (e.g., “slightly satisfied” vs. “very satisfied;” “matched expectations” vs. “below” or “above expectations”) and formulate meaningful answers. Because there were no issues with category discrimination, response categories were expanded from five to seven to allow for more options at the negative and positive ends of the scale.

Acknowledgements

We are grateful for the contribution of Theresa Carpenter and Megan Deng in collecting, coding, and entering the data of the cognitive interviews.

**References**

Baldwin, A. S., Rothman, A. J., & Jeffery, R. W. (2009). Satisfaction with weight loss: Examining the longitudinal covariation between people’s weight-loss-related outcomes and experiences and their satisfaction. *Annals of Behavioral Medicine*, *38*(3), 213–224. https://doi.org/10.1038/jid.2014.371

Baldwin, A. S., & Sala, M. (2018). Perceived satisfaction with health behavior change. In D. M. Williams, R. E. Rhodes, & M. T. Conner (Eds.), *Affective Determinants of Health Behavior* (pp. 69–89). Oxford University Press.

Barnes, D. C., & Krallman, A. (2019). Customer delight: A review and agenda for research. *Journal of Marketing Theory and Practice*, *27*(2), 174–195. https://doi.org/10.1080/10696679.2019.1577686

Jones, F., Harris, P., Waller, H., & Coggins, A. (2005). Adherence to an exercise prescription scheme: The role of expectations, self-efficacy, stage of change and psychological well-being. *British Journal of Health Psychology*, *10*(3), 359–378. https://doi.org/10.1348/135910704X24798

Ma, J., Scott, N., Gao, J., & Ding, P. (2017). Delighted or satisfied? Positive emotional responses derived from theme park experiences. *Journal of Travel and Tourism Marketing*, *34*(1), 1–19. https://doi.org/10.1080/10548408.2015.1125824

Neff, K., & King, A. C. (1995). Exercise program adherence in older adults: The importance of achieving one’s expected benefits. *Medicine and Exercise in Nutrition and Health*, *4*, 355–362.

Rothman, A. J. (2000). Toward a theory-based analysis of behavioral maintenance. *Health Psychology*, *19*(11), 64–69.

Sears, S. R., & Stanton, A. L. (2001). Expectancy-value constructs and expectancy violation as predictors of exercise adherence in previously sedentary women. *Health Psychology*, *20*(5), 326–333. https://doi.org/10.1037/0278-6133.20.5.326

Wilcox, S., Castro, C. M., & King, A. C. (2006). Outcome expectations and physical activity participation in two samples of older women. *Journal of Health Psychology*, *11*(1), 65–77. https://doi.org/10.1177/1359105306058850

Williams, D. M., Dunsiger, S., Davy, B. M., Kelleher, S. A., Marinik, E. L., & Winett, R. A. (2016). Psychosocial mediators of a theory-based resistance training maintenance intervention for prediabetic adults. *Psychology and Health*, *31*(9), 1108–1124. https://doi.org/10.1080/08870446.2016.1179740

Williams, D. M., Lewis, B. A., Dunsiger, S., Whiteley, J. A., Papandonatos, G. D., Napolitano, M. A., Bock, B. C., Ciccolo, J. T., & Marcus, B. H. (2008). Comparing psychosocial predictors of physical activity adoption and maintenance. *Annals of Behavioral Medicine*, *36*(2), 186–194. https://doi.org/10.1007/s12160-008-9054-7

Willis, G. B. (2004). *Cognitive interviewing: A tool for improving questionnaire design*. SAGE Publications.

Section D.

Data Screening and Preparation

Missing gender information at a given time point was imputed based on available data (e.g., gender information provided at T_2_ was imputed for missing gender information at T_1_). Prior to conducting the invariance analyses, a visual inspection of the responses to the enjoyment and satisfaction items indicated that distributions were slightly negatively skewed (see below for example distributions). Additionally, a comparison was made between those who responded to the survey at both times and those who dropped out after completing the survey at T_1_. Dropouts and completers were similar on demographic characteristics, mean responses to the enjoyment and satisfaction items, and frequency of leisure-time physical activity (see Supplementary Table D.1.).

Supplementary Figure D.1. Example Distribution of the First Item of the Physical Activity Enjoyment Scale-8 (PACES-8) Measure at T_1_.

Supplementary Figure D.2. Example Distribution of the First Item of the Physical Activity Enjoyment Scale-8 (PACES-8) Measure at T_2_.

Supplementary Figure D.3. Example Distribution of the First Item of the Satisfaction Measure at T_1_.

Supplementary Figure D.4. Example Distribution of the First Item of the Satisfaction Measure at T_2_.Supplementary Table D.1. Comparison of Time 1 Demographic Information and Scores on the First Items of the Satisfaction and Enjoyment Measures for Completers and Dropouts

| **Variable** | **Completers**  **(*n* = 314)** | **Dropouts**  **(*n* = 96)** | **Effect Size** |
| --- | --- | --- | --- |
|  |  |  |  |
|  | **Mean (SD)** | | **SMD (95% CI)** |
| Age (years) | 66.40 (7.21) | 65.97 (6.51) | 0.06 (-0.17, 0.30) |
| BMI (kg/m^2^) | 28.04 (5.82) | 27.45 (4.96) | 0.11 (-0.12, 0.35) |
| Physical activity | 5.54 (1.81) | 5.81 (1.47) | -0.18 (-0.41, 0.05) |
| Education | 5.20 (1.53) | 5.11 (1.46) | 0.06 (-0.18, 0.30) |
| Evaluation | 5.26 (1.58) | 5.11 (1.51) | 0.10 (-0.13, 0.33) |
| Pleasurable | 5.52 (1.45) | 5.33 (1.32) | 0.14 (-0.09, 0.37) |
|  |  |  |  |
|  | **Percentage** | | **OR (95% CI)** |
| White/Caucasian | 92.8% | 86.8% | 0.51 (0.24, 1.08) |
| Married/partnership | 65.7% | 61.5% | 0.84 (0.52, 1.36) |
| Retired | 55.4% | 60.0% | 1.21 (0.75, 1.95) |
| “Excellent” or “very good” self-rated health | 48.9% | 57.1% | 1.40 (0.87, 2.24) |

*Notes*. BMI: body mass index. SD: standard deviation. SMD: standardized mean difference. OR: odds ratio. CI: confidence interval. Education (highest degree received) ranged from 1 (some high school, no diploma) to 8 (doctorate).

Section E.

Additional Participant Demographics, Descriptive Statistics, and Omega Coefficients

Supplementary Table E.1. Participant Demographic Characteristics Reported at Baseline (T_1_; *n* = 392)

| Demographic Characteristic | Percentage (%) |
| --- | --- |
| Female | 48.0 |
| White/Caucasian | 88.0 |
| Married/domestic partnership | 63.8 |
| Retired | 54.3 |
| Bachelor’s degree or higher | 72.7 |
| “Excellent” or “very good” self-rated health | 50.2 |
| Arthritis | 28.8 |
| Heart disease | 14.3 |

Supplementary Table E.2. Means and Standard Errors (SE) for the PACES-8 and Satisfaction Items at T_1_ and T_2_

| **Item No.** | **Items** | **First measurement occasion (T_1_)** | | **Second measurement occasion (T_2_)** | |
| --- | --- | --- | --- | --- | --- |
|  |  | **Mean** | **SE** | **Mean** | **SE** |
|  | *Enjoyment* |  |  |  |  |
| 1 | Pleasurable | 5.55 | 0.07 | 5.59 | 0.08 |
| 2 | Fun | 5.12 | 0.07 | 5.11 | 0.08 |
| 3 | Pleasant | 5.31 | 0.07 | 5.39 | 0.08 |
| 4 | Invigorating | 5.33 | 0.07 | 5.29 | 0.08 |
| 5 | Gratifying | 5.65 | 0.07 | 5.66 | 0.08 |
| 6 | Exhilarating | 4.81 | 0.07 | 4.84 | 0.09 |
| 7 | Stimulating | 5.25 | 0.07 | 5.21 | 0.08 |
| 8 | Refreshing | 5.33 | 0.07 | 5.35 | 0.08 |
|  | *Satisfaction* |  |  |  |  |
| 1 | Evaluation | 5.34 | 0.08 | 5.23 | 0.09 |
| 2 | Expectations | 3.99 | 0.07 | 3.93 | 0.08 |
| 3 | Realizations | 5.28 | 0.08 | 5.21 | 0.09 |
| 4 | Emotion | 5.31 | 0.07 | 5.30 | 0.08 |

*Notes*. Responses to the PACES-8 items range from 1 to 7; higher values indicate higher levels of enjoyment of physical activity. Responses to the satisfaction items range from 1 to 7; higher values indicate higher levels of satisfaction with physical activity.Supplementary Table E.3. Omega Coefficients at T_1_ and T_2_ for Men and Women

| Measure | First measurement occasion (T_1_) | | | Second measurement occasion (T_2_) | | |
| --- | --- | --- | --- | --- | --- | --- |
|  | *All* | *Men* | *Women* | *All* | *Men* | *Women* |
| *Enjoyment* | 0.94 | 0.94 | 0.94 | 0.94 | 0.95 | 0.93 |
| *Satisfaction* | 0.90 | 0.90 | 0.89 | 0.91 | 0.91 | 0.90 |
